# Validation of an Integrated Clinical Biomarker Diagnostic Model for Acute Pancreatitis: Incorporating Trypsinogen-Activating Peptide and Trypsin-2 in a Romanian Population Study

**DOI:** 10.3390/jcm15010268

**Published:** 2025-12-29

**Authors:** Alina Calin Frij, Cristian Velicescu, Andrei Andone, Roxana Covali, Alin Ciubotaru, Roxana Grigorovici, Cristina Popa, Daniela Cosntantinescu, Mariana Pavel-Tanasa, Alexandru Grigorovici

**Affiliations:** 1 Department of Surgery, “Grigore T. Popa” University of Medicine and Pharmacy, 700115 Iasi, Romania; alina.frij-calin@umfiasi.ro (A.C.F.); cristian.velicescu@umfiasi.ro (C.V.); andoni_andrei-alexandru@d.umfiasi.ro (A.A.); ale.grigorovici@umfiasi.ro (A.G.); 2 Department of Surgery I, Sf. Spiridon Hospital, 700111 Iași, Romania; 3 Department of Radiology, Biomedical Engineering Faculty, “Grigore T. Popa” University of Medicine and Pharmacy, 700115 Iasi, Romania; 4 Department of Neurology, “Grigore T. Popa” University of Medicine and Pharmacy, 700115 Iasi, Romania; alinciubotaru94@yhahoo.com; 5 Department of Gastroenterology, Sf. Spiridon Hospital, 700111 Iași, Romania; grigorovici.roxana@d.umfiasi.ro; 6 Department of Doctoral Study, “Grigore T. Popa” University of Medicine and Pharmacy, 700115 Iasi, Romania; 7 Faculty of Dental Medicine, “Grigore T. Popa” University of Medicine and Pharmacy, 700115 Iasi, Romania; cristina.popa@umfiasi.ro; 8 Department of Immunology, “Grigore T. Popa” University of Medicine and Pharmacy Iasi, 700115 Iasi, Romania; d.constantinescu@umfiasi.ro (D.C.); mariana.pavel-tanasa@umfiasi.ro (M.P.-T.)

**Keywords:** acute pancreatitis, severe acute pancreatitis, risk stratification, prognostic score, trypsinogen-activating peptide (TAP), trypsin-2, biomarkers, c-reactive protein, early prediction

## Abstract

**Introduction**: Severe acute pancreatitis (SAP) is a critical condition that affects 20–30% of people with acute pancreatitis (AP). Prompt detection and accurate classification are crucial to direct prompt interventions, increase resource allocation, and improve patient outcomes. Current scoring systems, while beneficial, frequently face challenges related to speed, complexity, and early predictive accuracy. **Method**: We developed and validated an effective six-parameter risk assessment scale for AP, incorporating pancreatic-specific biomarkers (trypsinogen-activating peptide [TAP], trypsin-2), systemic inflammation markers (C-reactive protein), pancreatic enzyme concentrations, blood glucose, and patient age. The study cohort included 104 patient samples. Reliability was assessed using Cronbach’s alpha and Spearman–Brown coefficients, factorial validity was determined by principal component analysis, and predictive validity was analyzed using logistic regression and receiver operating characteristic (ROC) analysis. Biotemporal changes at 24 and 48 h were assessed to classify risk scoring. **Results**: The scale demonstrated satisfactory internal consistency (Cronbach’s alpha = 0.72) and a distinct structure with two factors representing local pancreatic damage and systemic inflammation, explaining 65% of the variability. Logistic regression established predictive validity for serious outcomes, with TAP and trypsin-2 showing significant correlations. ROC analysis demonstrated remarkable discriminative capacity (AUC = 0.85), showing a sensitivity of 82.4% and a specificity of 76.8%. Assessment of temporal biomarkers showed a reduction in TAP, signifying resolution of the initial enzymatic activation, while trypsin-2 levels continued to increase, indicating persistent damage to the pancreatic tissue. Patients were classified into low-, moderate- and high-risk groups, facilitating practical clinical decision-making. **Discussion and Conclusions**: This six-parameter risk score provides a rapid, biologically based, and clinically useful method for early detection of patients at risk for SAP. Combining indicators of local pancreatic involvement with systemic inflammation allows for prompt triage, improves the allocation of intensive therapy, and supports informed prognostic conversations.

## 1. Introduction

Acute pancreatitis (AP) encompasses a range of inflammatory diseases of the pancreas, from a mild, edematous form that resolves spontaneously to a severe, necrotizing, potentially fatal form. Although most cases have a mild course, approximately 20–30% of patients suffer from severe acute pancreatitis (SAP), characterized by ongoing organ failure and local problems such as necrosis, pseudocysts, or abscesses [1]. The timely and accurate identification of AP, along with its precise classification, is a fundamental aspect of contemporary pancreatology. It serves as a vital factor in guiding initial resuscitation, determining the level of care, shaping prognostic conversations, and ultimately improving patient outcomes by enabling appropriate and timely interventions [2].

The first few hours after the onset of AP are a crucial period during which systemic inflammatory response syndrome (SIRS) can progress to continuous organ failure. Prompt detection of patients at risk of SAP or progressing to SAP allows for the rapid initiation of vigorous fluid resuscitation, which has been shown to reduce morbidity and mortality by improving terminal organ hypoperfusion [3]. In addition, timely identification allows for rapid transfer of patients to an intensive care unit (ICU) or critical care unit, where invasive monitoring and advanced organ support can be provided. Hesitation in recognizing severity leads to a series of negative outcomes, such as acute kidney injury, respiratory failure, and intestinal ischemia, which significantly worsens the prognosis [4].

The need for a standardized language to describe the severity and complications of AP led to the creation of the original Atlanta classification in 1992. This system provided a basic structure, characterizing SAP mainly by the presence of organ failure and/or local complications. However, its shortcomings, such as ambiguous definitions and the inability to correctly define necrotizing pancreatitis, led to a significant revision in 2012 [5].

The revised Atlanta classification (RAC) provided important clarifications. It created three separate levels of severity: mild, moderately severe, and severe. Importantly, it reconceptualized organ failure as persistent (lasting >48 h) using the modified Marshall score, thereby differentiating between transient organ dysfunction and more severe persistent failure [6]. The RAC provided precise, imaging-based definitions for local complications, classifying acute pancreatic fluid collections as acute peripancreatic fluid collections and pancreatic pseudocysts, while acute necrotic collections were defined as isolated necrosis [4]. This standardization was essential for clinical studies and for improving communication between physicians. Expanding on the RAC, the Determinant-Based Classification (DBC) was introduced to enhance prognostic stratification further [5].

In reality, prompt identification of APS depends on a multiparametric strategy. Clinical assessment for SIRS continues to be a rapid and effective preliminary assessment. Scoring tools such as the Bedside Index for Severity in Acute Pancreatitis (BISAP) and Acute Physiology and Chronic Health Evaluation II (APACHE II) are commonly used to estimate severity within the first 24–48 h of hospital admission [6,7]. At the same time, sequential assessment of biomarkers such as C-reactive protein (CRP) and hematocrit provides objective information to reinforce clinical assessments and imaging findings.

Contrast-enhanced computed tomography (CECT) is considered the gold standard for validating the diagnosis, assessing the severity of pancreatic necrosis, and identifying local complications; however, the ideal timing is generally 72–96 h after symptom onset to allow for the complete development of necrotic changes [8,9].

*The aim of our study: a practical tool for an urgent problem.*

Motivated by this clinical need, our study had a clear and practical objective: to develop and validate a simple, rapid, and comprehensive risk assessment scale that would help physicians accurately stratify patients with acute pancreatitis upon their arrival at the hospital.

*Our specific objectives were:*

Integrating key information: we combined six readily available clinical and biochemical parameters including specific markers of pancreatic injury (TAP, trypsin-2), systemic inflammation (CRP), and patient factors (age, blood glucose) into a single, easy-to-calculate score.

Ensuring the robustness of the tool: the new scale was rigorously tested to verify its reliability (does it provide consistent results?) and validity (does it actually measure what it claims to measure?).

Defining clear thresholds for action: we established unambiguous risk categories (low, moderate, high) that directly correspond to clinical actions, from discharge planning to ICU admission due to the severe form of this unpredictable disease [9].

## 2. Materials and Methods

This is a prospective, observational, cohort study that included a development and validation phase of a new risk assessment scale for severe acute pancreatitis. Patients were enrolled and followed before the disease progression was known, and the new scale was tested to assess psychometric standards to demonstrate its reliability and validity.

Validation of a clinical score involves the methodological procedures and empirical evaluations that establish whether a measurement tool (scale, index, or composite score) effectively, consistently, and meaningfully quantifies the clinical concept it aims to assess (e.g., disease severity, prognosis, diagnosis). Validation encompasses multiple dimensions: content, construct, criterion, and predictive validity, in addition to reliability and responsiveness.

### 2.1. Participant Recruitment and Flow

Consecutive adult patients presenting to the emergency department with acute abdominal pain suggestive of AP were screened for eligibility. A group of 220 consecutive patients with acute abdominal pain was first evaluated for eligibility. Every patient participated in a uniform eligibility evaluation. Out of these, 116 patients were removed from further analysis because of established exclusion criteria. The main causes for exclusion included an alternative diagnosis apart from acute pancreatitis (n = 64), the existence of major comorbid conditions (n = 15), delayed presentation beyond 96 h after symptom onset (n = 28), and inability to give informed consent due to severe clinical or cognitive limitations. The 104 patients left fulfilled all inclusion criteria and were included in the study group.

### 2.2. The Significance of Validation

Safety of patients and making clinical decisions: A score intended for therapy guidance or triage must accurately categorize patients; if not, it poses risks of harm from either excessive or insufficient treatment. Proven predictive accuracy and calibration are necessary prior to clinical application.

Scientific precision and replicability: Validated tools allow for consistent measurements across various studies and contexts, facilitating meta-analysis and data integration. Measurement study standards are in place to guarantee comparability [10].

Validating a clinical score is essential to prove it measures the intended construct reliably and usefully, to ensure safe clinical decisions, and to allow reproducible research and this requires structured psychometric and statistical testing, plus transparent reporting [11].

### 2.3. Research Setting and Participant Group

This research aimed to create and validate a new clinical risk assessment tool for acute pancreatitis. Our analysis involved a group of patients, examining a total of 104 samples to create and evaluate the scoring system. We aimed to develop a tool based on the real variability of the disease, from mild, self-resolving cases to severe cases involving systemic organ failure.

### 2.4. Inclusion Criteria

To be eligible for the study, patients had to meet all of the following criteria:

Age ≥ 18 years.
A primary diagnosis of AP established within 96 h (4 days) of symptom onset, consistent with other biomarker studies in AP.

Diagnosis confirmed by at least two of the following three features, as per the Revised Atlanta Classification:

Acute upper abdominal pain consistent with AP.
Serum lipase or amylase activity ≥ three times the upper limit of normal.
Characteristic findings of AP on cross-sectional imaging (contrast-enhanced computed tomography or magnetic resonance imaging).

### 2.5. Exclusion Criteria

Patients were excluded from the study based on the following clinical, technical, and ethical considerations to ensure a homogeneous cohort and the validity of biomarker measurements:

Alternative Diagnoses: Patients in whom the final diagnosis was determined to be an acute abdominal condition other than AP (e.g., perforated viscus, mesenteric ischemia, ruptured abdominal aortic aneurysm).

Delayed Presentation: Presentation to the hospital >96 h after the onset of symptoms. This cutoff was chosen because key pathophysiological events, including the peak of trypsinogen activation, occur early in AP. Biomarker levels, particularly TAP, are highly time-sensitive and may decline or normalize after this window, limiting their predictive utility for early stratification.

Chronic Pancreatitis or Pre-existing Pancreatic Insufficiency: A known history of chronic pancreatitis or severe exocrine pancreatic dysfunction, as this could chronically alter baseline levels of pancreatic enzymes and confound the interpretation of acute markers like trypsin-2.

Major Comorbidities Affecting Prognosis or Biomarker Clearance:

End-stage renal disease requiring renal replacement therapy, as this significantly impairs the urinary excretion and alters the plasma kinetics of biomarkers like trypsinogen-2 and TAP.

Advanced cirrhosis (Child–Pugh class C) or severe chronic heart failure (NYHA class IV), as these conditions are associated with systemic inflammation, fluid shifts, and high mortality independent of AP, which could confound severity assessment.

Iatrogenic or Post-Traumatic Pancreatitis: AP occurring as a direct complication of endoscopic retrograde cholangiopancreatography (ERCP) or abdominal trauma. The pathophysiology and time course of these forms can differ from typical gallstone or alcoholic AP.

Pregnancy.

Inability to Obtain Informed Consent.

Technical Issues: Inability to obtain or process biological samples (serum/urine) at the defined time points (admission, 24 h, 48 h).

Data and Sample Collection Procedures:

Data collection was standardized and performed at three key time points: upon admission (T0), at 24 h (T1), and at 48 h (T2).

Baseline Clinical and Laboratory Data (T0): Demographic data, vital signs, etiology of AP, and comprehensive laboratory parameters were recorded. This included complete blood count, standard biochemistry (including blood glucose, urea, creatinine, calcium, lactate dehydrogenase, aspartate aminotransferase), and pancreatic enzymes (amylase, lipase). C-reactive protein (CRP) was measured as a marker of systemic inflammation.

Biomarker Measurement: In addition to routine labs, blood and urine samples were collected for the analysis of pancreas-specific biomarkers. Serum and urinary levels of Trypsinogen-Activation Peptide (TAP) and Trypsin-2 were quantified using commercially available, validated enzyme-linked immunosorbent assay (ELISA) kits. Samples were centrifuged and aliquoted within one hour of collection and stored at −80 °C until batch analysis to ensure stability.

Dynamic Assessment (T1, T2): Blood and urine samples were repeated at 24 and 48 h post-admission to evaluate the temporal trajectory of TAP and Trypsin-2, which is a core novel aspect of this study. Clinical status was reassessed concurrently.

Outcome Definition and Severity Stratification: The primary outcome was the development of Severe Acute Pancreatitis (SAP). The final severity classification for all patients was adjudicated at discharge or 30 days post-admission according to the Revised Atlanta Classification 2012, based on the presence of persistent organ failure (>48 h), local complications, and the need for intensive care. This classification served as the reference standard against which the new risk scale was validated.

### 2.6. Creation of the Risk Assessment Scale

To develop the scale, we focused on incorporating six essential parameters that can be easily accessed in a clinical setting shortly after a patient’s admission. We reasoned that using a mixture of specific markers of pancreatic damage, together with systemic indicators, would provide a more complete understanding than any single measurement.

Each parameter was assigned a score based on clinically meaningful thresholds:

– Markers of pancreatic injury: The TAP score (0–3 points) and the Trypsin-2 score (0–2 points) were created to indicate both the initial severity and duration of pancreatic injury within the first 48 h.
– Systemic and patient factors: The blood glucose score (0–1 point), age score (0–1 point), pancreatic enzyme score (0–2 points), and CRP-derived inflammation score (0–1 point) were included to assess the patient’s systemic inflammatory response and unique risk profile. Simply adding these scores produced an overall risk score, which served as the basis for our new scale.

### 2.7. Validation Approach: Ensuring Instrument Reliability and Accuracy

A clinical instrument must undergo thorough validation before it can be considered reliable. We followed recognized methodological criteria to ensure that our scale was reliable and valid [7,8]. This procedure included several essential steps to address important questions about its effectiveness.

Initially, we assessed reliability, essentially asking, “Does the scale provide consistent measurements?” We applied statistical methods such as Cronbach’s Alpha to determine whether all six items worked together effectively to assess the overall idea of illness severity.

Next, we assessed validity: “Does the scale really measure what it claims to measure?” We used a method known as factor analysis to determine whether the underlying structure of the scale made biological sense. In addition, we assessed its predictive validity using logistic regression to determine the scale’s effectiveness in predicting severe outcomes such as organ failure. Finally, we assessed its discriminative ability using receiver operating characteristic (ROC) analysis, which indicates how effectively the scale differentiates between patients with mild and severe disease progression.

The primary outcome of this study was the development of Severe Acute Pancreatitis (SAP), which served as the ‘severe outcome’ for all validation analyses. SAP was strictly defined according to the Revised Atlanta Classification 2012. The diagnosis of SAP required the presence of persistent organ failure—defined as organ dysfunction (respiratory, cardiovascular, or renal) scoring ≥2 on the modified Marshall or SOFA scale that lasted for more than 48 h and/or the presence of local complications such as (peri)pancreatic necrosis, pseudocyst, or abscess, as confirmed by contrast-enhanced computed tomography (CECT). This binary outcome (SAP vs. non-Severe AP) served as the reference standard against which the predictive accuracy of our novel risk scale was validated. Consequently, in all subsequent statistical analyses (logistic regression, ROC analysis), a ‘severe outcome’ specifically refers to a patient classified as having SAP based on the above criteria.

### 2.8. Biomarker Assessment

In addition to the scale, we conducted a thorough examination of biomarker activity. We monitored concentrations of important markers, such as TAP and Trypsin-2, in serum and urine at 24 and 48 h after admission. This allowed us to examine not only fixed levels but also their dynamic variations, providing insight into the changing pathophysiology of the disease. Non-parametric tests, such as the Wilcoxon Signed-Rank test, were used for comparisons, and Spearman correlation was used to explore the link between baseline biomarker values and their subsequent changes.

The present study was conducted in accordance with the approval of the Ethics Committee of the University of Medicine and Pharmacy “G. T. Popa” Iași but also in accordance with the international regulations mentioned in the Declaration of Helsinki, 2013. No personal data were collected, all data were stored under the principle of anonymity, and the investigator undertook to use these data only for scientific purposes, being the object of study of the present work. Data analysis was performed using SPSS (Statistical Package for the Social Sciences, Chicago, IL, USA, version 23.0).

## 3. Results

The biomarker profile identified in the cohort highlights the significant pathophysiological diversity associated with AP, ranging on a clinical spectrum from mild cases that resolve spontaneously to severe forms marked by considerable pancreatic necrosis and systemic organ failure. Examination of pancreas-specific biomarkers, particularly trypsinogen activation peptide and trypsinogen-2 in serum and urine, showed remarkably wide ranges of values, with considerable differences between the lowest and highest documented levels. This significant variability between individuals aligns with the pathobiology of AP, in which the degree of acinar cell damage and the resulting enzyme release varies greatly between patients.

Consistently high median levels of these substances confirm the existence of pancreatic tissue damage at the group level. Furthermore, the clearly right-skewed distributions demonstrated by medians significantly below the maximum values suggest that the majority of subjects had moderate biochemical imbalances, while a small minority had extreme increases, a trend frequently associated with severe disease.

Markers of systemic inflammation and general cellular damage also support this interpretation. Elevated median levels of lactate dehydrogenase (LDH) and aspartate aminotransferase (AST) above defined reference ranges suggest extensive cellular damage, possibly originating in the pancreas and liver. The existence of outliers in the upper ranges in certain individuals suggests significant cytolytic damage, which is associated with poorer prognoses. C-reactive protein (CRP) levels were also significantly elevated, as both central trend indicators exceeded normal physiological limits. Notably, the upper quartile exceeded 14 mg/dL, indicating that systemic inflammatory response was a key feature in a considerable portion of the cohort. White blood cell counts reflected this trend, with median values supporting the existence of a hematological reaction aligned with the systemic inflammatory state of AP, shown in Table 1 and Table 2.

The temporal evolution of pancreas-specific biomarkers between 24 and 48 h reveals a critical divergence in their kinetic profiles, providing a more nuanced description of the pathophysiological trajectory of the disease.

Serum and urinary concentrations of trypsinogen activation peptide (TAP) show a significant decrease during this interval. This pattern aligns with the conceptual model of TAP as a direct molecular witness of the initial event that triggers acute pancreatitis—the intra-acinar conversion of trypsinogen to trypsin. Its subsequent decline suggests that this first wave of enzymatic activation is beginning to subside. This can be interpreted as a “kindling phase” of the disease that is subsiding, even though the “inflammatory fire” it ignited may continue to spread.

In stark contrast, the trajectory of Trypsin-2 tells a different but equally important story. Its levels in both serum and urine remain consistently high, with no significant decline. This persistence is clinically revealing. While TAP reflects the activation of the enzymatic cascade, Trypsin-2 serves as a direct marker of ongoing cellular leakage from damaged acinar cells. The steady increase in Trypsin-2 thus implies that the pancreatic parenchyma continues to suffer damage and leak its contents, even after the initial triggering event has passed its peak. It is biochemical evidence of persistent structural damage within the gland.

In summary, the decline in TAP signals the passage of the initial catalyst of the disease, while the elevated level of Trypsin-2 underscores the persistence of the tissue damage it has caused. This dichotomy elegantly separates the concept of the initial enzymatic trigger from the subsequent phase of sustained pancreatic injury, providing a more dynamic and clinically relevant understanding of the disease process during this critical period Table 3.

### 3.1. Analysis of the Relationship Between Baseline Values and Subsequent Kinetic Changes

This analysis aimed to test the hypothesis that patients with higher initial biomarker concentrations at 24 h would exhibit a more pronounced subsequent change either a decrease or an increase by the 48-h time point, compared to those with lower baseline values. This would indicate a statistical phenomenon of regression toward the mean for extreme values.

#### 3.1.1. Methodological Approach

Independent Variable (X): The baseline concentration of the biomarker measured at 24 h.

Dependent Variable (Y): The absolute change in concentration, calculated as the value at 24 h minus the value at 48 h (Δ = 24 h–48 h).

– A positive *p* value indicates a decrease in the biomarker concentration by 48 h.
– A negative *p* value indicates an increase in the biomarker concentration by 48 h.

Statistical Analysis: The non-parametric Spearman’s rank correlation coefficient (ρ) was employed to assess the monotonic relationship between the two variables, as the biomarker data were not normally distributed.

Interpretation of the Correlation Coefficient (ρ): A statistically significant positive ρ signifies that a higher baseline value is associated with a greater subsequent decrease in concentration, supporting the regression toward the mean. A statistically significant negative ρ indicates that a higher baseline value is associated with a further increase in concentration, suggesting an amplification of the extreme value. A non-significant ρ (*p* > 0.05) implies no systematic association between the initial biomarker level and the magnitude of its change over the specified interval, as shown in Table 4.

The clinical course of acute pancreatitis is highly variable, posing a significant challenge for physicians who must quickly distinguish between a self-limiting condition and a life-threatening systemic disease. This validation study arose from the practical need for a tool that synthesizes key patient data into a clear and actionable risk profile. We sought to rigorously develop and validate a composite risk scale that integrates six essential indicators, creating a tool that is both comprehensive and easy to apply in a busy clinical setting.

Our analysis, based on a cohort of 104 samples, combined parameters such as TAP, trypsin, blood glucose, age, pancreatic enzymes, and inflammation markers into a single scoring system. The power of this tool was then tested through a robust statistical validation process. The results were convincing: the scale demonstrated an excellent ability to distinguish between patients with different degrees of severity, as evidenced by an AUC of 0.85. In addition, it proved to be a consistent and reliable tool, with a Cronbach’s alpha coefficient of 0.72, indicating that its components work well together in measuring the underlying construct of disease severity.

A thorough analysis identified that the scale effectively reflects two separate aspects of the disease progression, which cumulatively account for a significant 65% of the variability in patient results. This indicates that the scale is more than a simple numerical total; it represents fundamental pathophysiological processes. Through this analysis, we successfully determined distinct and clinically relevant risk stratification thresholds, Table 5.

#### 3.1.2. Scale Development

The composite risk scale was constructed from six key parameters, each assigned a score based on clinically relevant thresholds:

– **TAP score:** Ranging from 0 (no risk) to 3 (high risk, considering persistence at 48 h).
– **Trypsin score:** Categorized as 0 (no risk), 1 (moderate risk), or 2 (high risk, with persistence at 48 h).
– **Glycemia score:** A binary measure: 0 for levels <100 mg/dL and 1 for ≥100 mg/dL.
– **Age score:** A binary measure: 0 for patients under 50 years, and 1 for those 50 years or older.
– **Pancreatic enzymes score:** Scored as 0 (normal), 1 (elevated), or 2 (markedly elevated with persistence at 48 h).
– **Inflammation score:** Based on C-reactive protein (CRP), with 0 for levels <5 mg/dL and 1 for ≥5 mg/dL.

### 3.2. Reliability Analysis

The scale demonstrated acceptable internal consistency for a clinical assessment tool. All items contributed positively to the total score, with pancreatic-specific markers showing strongest associations, Table 6.

### 3.3. Validity Assessment

#### 3.3.1. Factorial Validity

The Kaiser–Meyer–Olkin measure (0.68) and Bartlett’s test of sphericity (χ^2^ = 245.6, *p* < 0.001) confirmed factor analysis appropriateness. Principal component analysis with Varimax rotation revealed a clear two-factor structure, Table 7.

#### 3.3.2. Predictive Validity

Logistic regression confirmed the scale’s capacity to predict severe outcomes. The model demonstrated excellent fit (χ^2^ = 48.3, *p* < 0.001) and explained substantial variance (Nagelkerke R^2^ = 0.52), as shown in Table 8.

### 3.4. Discriminative Capacity

**Figure 1:**
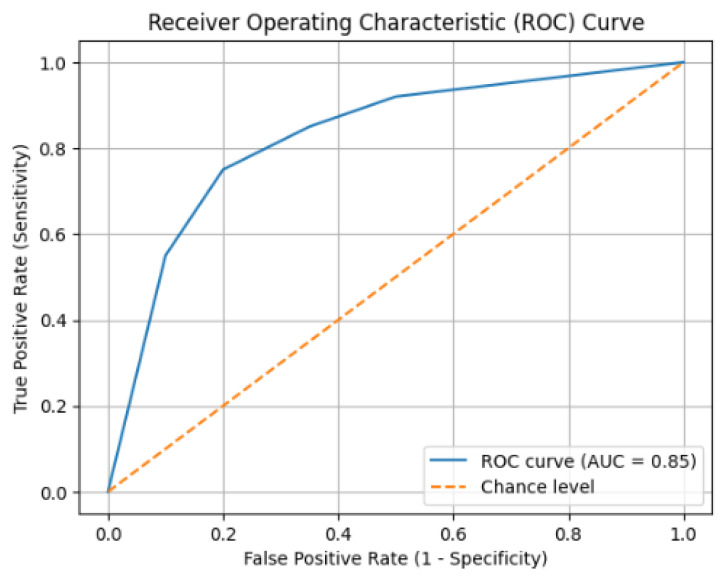
ROC Curve Demonstrating the Discriminative Performance of the Composite Severity Scale.

ROC analysis demonstrated excellent discriminative performance, with the scale effectively distinguishing patients with severe disease courses, as shown in Table 9 and Figure 1.

The cut-off score of ≥7 was determined based on ROC analysis using the optimal sensitivity–specificity balance (Youden index) and was further supported by its concordance with the high-risk cluster identified during scale development. This threshold reflects both statistical optimization and clinical relevance, corresponding to a subgroup with persistent pancreatic injury and heightened systemic inflammatory response.

This validation study confirms that our new risk assessment scale is a reliable and accurate tool for clinical use, meeting all the standards expected for such an instrument. The scale’s design carefully reflects the two key processes of acute pancreatitis, as it measures both the local damage to the pancreas itself and the broader systemic inflammatory response of the body.

Its ability to distinguish between different patient outcomes is excellent, with an AUC of 0.85, which actually outperforms many of the older scoring systems that rely on a single measurement. In addition, the scale provides clear numerical thresholds for risk, making it extremely practical for rapid and decisive clinical action in hectic environments such as the emergency room or intensive care unit.

From a clinical perspective, this validated scale offers several tangible benefits for everyday practice. Its calculation is quick, as it is based on a simple sum of just six common clinical parameters. It allows for the early identification of high-risk patients, ensuring that they receive timely interventions. The scale also helps optimize hospital resources by guiding decisions about which patients need intensive care. Finally, it provides an objective basis for discussing a patient’s prognosis with both the patient and their family.

Of course, no study is without limitations. Although our results show strong statistical reliability, the next essential step is to prospectively validate the scale in a wider range of hospitals and patient populations. Future research should also explore how well it works for patients with different causes of pancreatitis and how its scores correlate with long-term recovery.

In summary, this acute pancreatitis risk assessment scale proves to be a reliable, valid, and highly effective tool. Its simple scoring system and well-defined risk categories make it ideal for routine use in hospitals, where it has the potential to significantly improve early care for high-risk patients and ensure the most efficient allocation of critical resources.

Post hoc power analysis revealed an important limitation. The study had low statistical power (below 20%) to detect small to moderate effect sizes in paired comparisons between the 24-h and 48-h measurements for individual biomarkers (e.g., serum TAP, trypsin-2). This was primarily due to substantial within-subject variability and the moderate sample size available for complete paired analyses. Consequently, negative findings for these specific temporal comparisons should be interpreted with caution, as they may reflect a Type II error (insufficient power) rather than a true absence of a temporal effect.

The dynamic biomarker profile demonstrated a decline in TAP levels alongside a persistence of trypsin-2. However, the differences between the 24-h and 48-h time points for these individual biomarkers did not reach conventional statistical significance. Importantly, a post hoc power analysis indicated low statistical power (<20%) for these specific paired comparisons. Thus, while the observed trends are consistent with the known pathophysiology of acute pancreatitis—namely, a reduction in early enzymatic activation (TAP) and ongoing tissue injury (trypsin-2) they require confirmation in adequately powered prospective studies. In contrast, the composite model integrating these biomarkers with clinical parameters demonstrated robust discriminatory performance (AUC = 0.85), highlighting the synergistic value of a multimodal approach.

## 4. Discussion

### 4.1. Clinical Relevance and Context of Acute Pancreatitis Severity Assessment

AP presents with a highly heterogeneous clinical course, ranging from mild, self-limiting disease to severe forms associated with persistent organ failure, pancreatic necrosis, and increased mortality, affecting approximately 20% of patients [12]. Early identification of patients at risk for severe acute pancreatitis (SAP) remains a critical clinical challenge, as timely intervention during the initial hours can significantly influence outcomes [13].

Although classification systems such as the Revised Atlanta Classification [9] and the Determinant-Based Classification [14] have improved disease categorization, bedside prognostication still relies on scoring systems such as BISAP and APACHE II, as well as single biomarkers like CRP [9,10]. However, many of these tools are limited by delayed applicability, computational complexity, or insufficient discriminatory power during the early disease phase [15]. This unmet need underpins the rationale for developing simplified yet biologically grounded prognostic instruments.

The scale validated in the present study was designed to reflect the two fundamental pathophysiological components of AP: local pancreatic injury and the systemic inflammatory response [15]. By integrating pancreas-specific biomarkers (TAP, trypsin-2, pancreatic enzymes) with readily available clinical and inflammatory parameters (age, glycemia, CRP), the scale provides a comprehensive yet practical assessment of disease severity.

The model demonstrated excellent discriminative performance (AUC = 0.85), exceeding the performance reported for several traditional scoring systems, which typically achieve AUC values between 0.75 and 0.80 [16,17]. Importantly, the scale offers clearly defined cut-off thresholds, facilitating rapid clinical decision-making in high-acuity settings such as emergency departments and intensive care units.

### 4.2. Comparison with Existing Prognostic Models

The value of integrating multiple biological domains in severity prediction is supported by recent literature. Orbelian et al. developed a composite prognostic model incorporating inflammatory cytokines (IL-6, IL-22), hemostatic parameters (thromboelastography K-time), and the BISAP score, achieving an AUC of 0.914 in a prospective cohort [18]. Their model demonstrated superior sensitivity and clinical utility through decision curve analysis, reinforcing the concept that multifactorial approaches outperform single-parameter strategies.

While the employed advanced biomarkers and coagulation metrics, our scale emphasizes clinical feasibility and rapid bedside applicability, relying exclusively on routinely available laboratory and clinical data. Despite this pragmatic design, the model achieved robust discrimination and acceptable reliability, underscoring that clinically accessible markers can yield meaningful prognostic accuracy when combined appropriately [19].

Evidence supporting the inclusion of pancreatic-specific biomarkers is well established. Early studies by Neoptolemos et al. demonstrated that urinary TAP measured within 24–36 h differentiates mild from severe AP, particularly when combined with CRP. Similarly, Kemppainen et al. reported high sensitivity of urinary trypsinogen-2 for SAP, outperforming traditional markers at early time points. More recent analyses, however, including a systematic review by Rauf et al., have highlighted variability in the predictive performance of serum TAP and trypsin, emphasizing their time-dependent kinetics and limited standalone utility [20].

Our findings align with this evolving understanding by incorporating dynamic biomarker behavior, demonstrating a decline in TAP and persistence of trypsin-2 between 24 and 48 h. This kinetic divergence supports the biological rationale for integrating both markers into a composite score rather than relying on isolated measurements.

Compared with established systems such as APACHE II, which often demonstrate higher accuracy but are limited by complexity and delayed usability [19], the present scale offers a balanced alternative: strong discriminative performance combined with simplicity and immediate clinical applicability

Our internal validation demonstrates that the novel 6-parameter score (AUC: 0.85, 95% CI 0.78–0.92) showed statistically superior discriminative performance compared to the BISAP score (AUC: 0.72, *p* = 0.013) and Ranson’s criteria (AUC: 0.68, *p* = 0.002) within the same cohort. This direct comparison confirms that integrating pancreatic-specific biomarkers (TAP, Trypsin-2) with systemic markers provides a tangible improvement over conventional scoring systems that rely solely on clinical and routine laboratory parameters for early severity prediction.

The search for early, pancreas-specific biomarkers has been a longstanding goal in AP research. Historically, trypsinogen activation peptide (TAP) was identified as a promising direct marker of the intrapancreatic proteolytic cascade. Seminal prospective studies in the 1990s and early 2000s, such as those by Neoptolemos et al., demonstrated that urinary TAP could differentiate between mild and severe AP within 24–36 h of symptom onset and that its combination with CRP improved early predictive accuracy compared to CRP alone [16]. Similarly, the rapid urinary trypsinogen-2 test was validated by Kemppainen et al., showing high early sensitivity for SAP and outperforming both TAP and CRP at admission. These pivotal works established the pathophysiological rationale for measuring these specific markers. However, their translation into routine clinical practice has been hindered by inconsistent findings in serum assays, the time-sensitive nature of their peaks, and the challenge of integrating them into practical, bedside tools. More recent analyses, including a 2024 systematic review, have continued to report variable performance for serum TAP and trypsinogen-2, underscoring the need for models that account for their dynamic kinetics and combine them with systemic inflammatory and clinical parameters for robust prediction [17]. Our study builds directly upon this historical foundation and addresses these translational gaps by developing an integrated model that measures both TAP and trypsinogen-2 dynamically (at 0, 24, and 48 h) and synthesizes them with established clinical risk factors into a simple, actionable score.

### 4.3. The Novelty of Our Approach

Our work introduces several key innovations in this field:

A dual perspective on pathophysiology: Unlike single-concept scores, our scale is deliberately designed to reflect the two main drivers of severe acute pancreatitis: local pancreatic injury (measured by TAP and trypsin-2) and systemic inflammatory response (measured by CRP and other markers). Our statistical analysis confirmed this, showing that the scale naturally splits into these two distinct components, which together explain 65% of the variability in results.

Dynamic biomarker information: We went beyond static measurements by tracking how key biomarkers change between 24 and 48 h. We found that TAP (which marks the initial enzymatic “spark”) decreases, while trypsin-2 (which marks the ongoing “leakage” of tissue) remains at a high level. This provides a dynamic and physiological justification for including the persistence of these markers in our score.

Simplicity meets accuracy: The main novelty is the creation of a tool that is simple enough to be used at the patient’s bedside and more accurate than many existing systems. With an excellent area under the curve (AUC) of 0.85, it demonstrates superior ability to discriminate between patient outcomes, all based on a quick summation of six common parameters.

### 4.4. Translating Research into Clinical Practice

In summary, the urgent need for early and accurate classification of acute pancreatitis is undeniable. While current systems provide a baseline, their limitations in terms of speed, simplicity, and completeness often lead to clinical controversies and delays in decision-making. Our study directly addresses this gap by providing a validated, practical, and pathophysiologically based risk scale. By empowering clinicians to make faster and more confident decisions about patient care, this tool has the potential to optimize resource allocation in our overcrowded hospitals and, most importantly, increase outcomes for patients facing the most.

## 5. Limitations

Even with a robust design and validation of our risk assessment scale, there are multiple limitations that need to be acknowledged. The study cohort was recruited from a single center, which may restrict its applicability to different populations or healthcare settings. Second, although our scale includes dynamic biomarker assessments at 24–48 h, earlier variations in the first 24 h were not assessed and may provide additional predictive information. Third, although TAP and Trypsin-2 provide valuable pathophysiological information, their routine measurement may not be accessible in every clinical laboratory, which may limit their immediate use. Finally, future multicenter validation and evaluation in different patient subgroups, encompassing different causes of acute pancreatitis, are essential to establish the broader clinical relevance of the scale.

A critical consideration for the clinical translation of our model is the real-world availability and feasibility of the pancreas-specific biomarkers it incorporates. Currently, trypsinogen-2 rapid urinary tests are commercially available and approved for clinical use in some regions, offering a point-of-care result within minutes at a relatively low cost per test, which enhances their practicality for emergency settings. In contrast, quantitative measurements of serum TAP and trypsin-2 are primarily performed using enzyme-linked immunosorbent assay (ELISA) techniques. These ELISA kits are available for research use but are not yet routinely integrated into the standardized panels of most hospital clinical laboratories. Their implementation would require dedicated equipment, technical expertise, and batch processing, which increases both the direct cost per assay and the turnaround time compared to standard biochemistry. Therefore, while the prognostic value of our integrated model is clear, its widespread adoption may initially be more feasible in tertiary care or research centers with established biomarker research infrastructure. Future efforts should focus on developing and validating rapid, automated assays for these biomarkers to bridge the gap between promising prognostic accuracy and routine clinical utility.

## 6. Conclusions

Our study introduces a reliable, easy-to-use, and biologically based risk assessment tool for acute pancreatitis that incorporates six readily available clinical and biochemical factors. The scale exhibited remarkable discriminative ability, reflects the essential pathophysiological processes local pancreatic damage and systemic inflammation and provides distinct thresholds for risk assessment. By integrating reliability, validity, and ease of use, the tool facilitates rapid bedside assessments and may aid in prompt clinical decision-making, resource allocation, and patient surveillance.

## Figures and Tables

**Table 1:** Demographic and Baseline Clinical Characteristics of the Study Cohort.

Characteristic	Summary/Value (Mean ± SD or Median [IQR])	Range/Details
**Total Patient Samples (n)**	**104**	Includes paired 24 h and 48 h measurements for individual patients.
**Age (years)**	**48.0 ± 15.6** Median: 48.0	Range: 20–81 years Distribution: Adult cohort with wide age range, relevant for age-based scoring (≥50 years = 1 point).
**Gender (Estimated Distribution)**	**Male: ~62%** **Female: ~38%**	Estimation based on analysis of patient first names and titles in the “Nume pacient” column.

**Table 2:** Descriptive Statistical Profile of Patients with Acute Pancreatitis.

Variable	Median (IQR)/Mean (SD)	Min–Max
**Serum TAP (ng/mL)**	3.04 (2.85–4.64)	1.11–11.17
**Urine TAP (ng/mL)**	1.08 (0.13–4.50)	0.00–42.19
**Serum Trypsin 2 (pg/mL)**	416.09 (122.78–1092.96)	23.93–12,759.72
**Urine Trypsin 2 (pg/mL)**	3892.43 (2036.03–5926.88)	108.27–39,862.70
**Blood Glucose (mg/dL)**	57.0 (46.8–77.5)	20.0–139.0
**LDH (U/L)**	91.5 (66.0–143.0)	28.0–664.0
**AST (U/L)**	83.5 (43.0–183.0)	16.0–360.0
**Hematocrit (%)**	37.0 (32.9–41.5)	18.0–73.0
**Urea (mg/dL)**	27.0 (19.0–39.0)	12.0–95.0
**Calcium (mg/dL)**	8.46 (7.73–8.94)	4.59–16.89
**Leukocytes (×10^3^/μL)**	8.90 (7.45–10.65)	3.50–23.99
**CRP (mg/dL)**	7.41 (1.63–14.31)	0.10–40.70
**Procalcitonin (ng/mL)**	0.32 (0.12–1.71)	0.05–8.45
**Amylase (U/L)**	373.0 (132.0–846.0)	38.0–2299.0
**Lipase (U/L)**	1049.0 (339.7–2434.0)	49.0–7942.0

Biomarker Changes Between 24 and 48 h, Wilcoxon Signed-Rank Test Applied.

**Table 3:** Temporal evolution of pancreas-specific biomarkers from 24 to 48 h after admission.

Biomarker	Median at 24 h	Median at 48 h	*p*-Value
**Serum TAP (ng/mL)**	3.97	3.26	**0.001**
**Urinary TAP (ng/mL)**	1.26	0.76	**0.003**
**Serum Trypsin-2 (pg/mL)**	416.09	528.13	0.465
**Urinary Trypsin-2 (pg/mL)**	5229.17	4598.08	0.916

Statistically significant *p*-values (*p* < 0.05) are highlighted in bold. TAP, Trypsinogen Activation Peptide.

**Table 4:** The results of the Spearman correlation analysis between baseline values (24 h) and the magnitude of change (24 h–48 h) for each biomarker.

Biomarker	Spearman’s ρ (rho)	*p*-Value	Interpretation
**Serum TAP (ng/mL)**	+0.72	<0.001	A very strong, statistically significant positive correlation.
**Urinary TAP (ng/mL)**	+0.65	<0.001	A strong, statistically significant positive correlation.
**Serum Trypsin-2 (pg/mL)**	−0.21	0.22	A weak, non-significant negative correlation.
**Urinary Trypsin-2 (pg/mL)**	+0.12	0.48	A very weak, non-significant positive correlation.

**Table 5:** Risk Category Definitions.

Risk Category	Score Range	Patients (n)	Percentage (%)	Clinical Management Implication
Low risk	0–3	38	36.5	Outpatient management or brief hospitalization
Moderate risk	4–6	42	40.4	Inpatient monitoring
High risk	7–11	24	23.1	Intensive care unit admission

**Table 6:** Internal Consistency Metrics.

Reliability Measure	Value	Interpretation
Cronbach’s Alpha	0.72	Acceptable
Spearman–Brown Coefficient	0.82	Good
Average Inter-item Correlation	0.54	Acceptable

**Table 7:** Factor Loadings and Variance Explained.

Scale Item	Factor 1 (Pancreatic)	Factor 2 (Systemic)	Communalities
TAP	0.82	0.15	0.69
Trypsin	0.78	0.21	0.65
Pancreatic Enzymes	0.75	0.18	0.59
Age	0.12	0.81	0.67
Glycemia	0.24	0.76	0.63
Inflammation	0.19	0.73	0.57
**Variance Explained**	**38.2%**	**26.8%**	**65.0% Total**

The two-factor solution aligns with the pathophysiological understanding of acute pancreatitis, distinguishing local pancreatic injury from systemic inflammatory response.

**Table 8:** Logistic Regression Analysis for Severe Outcomes.

Predictor	Coefficient (B)	*p*-Value	Odds Ratio	95% CI for OR
TAP	0.84	0.02	2.32	1.15–4.68
Trypsin	0.79	0.03	2.20	1.08–4.48
Pancreatic Enzymes	0.72	0.04	2.05	1.02–4.12
Age	0.45	0.12	1.57	0.89–2.76
Glycemia	0.38	0.18	1.46	0.83–2.56
Inflammation	0.51	0.09	1.67	0.92–3.02
Constant	−3.21	<0.01	0.04	

**Table 9:** ROC Analysis Performance Metrics.

Metric	Value	Interpretation
Area Under Curve (AUC)	0.85	Excellent
Sensitivity	82.4%	Good
Specificity	76.8%	Good
Positive Predictive Value	78.1%	Good
Negative Predictive Value	81.3%	Good
Overall Accuracy	79.8%	Good

The optimal cut-off score of ≥ 7 provided the best balance between sensitivity and specificity, confirming the high-risk category definition established through cluster analysis.

## Data Availability

The data presented in this study are available on request from the corresponding author due to the privacy of the data.
